# Editorial: Nutritional and adaptive aspects of ion transport in plants

**DOI:** 10.3389/fpls.2026.1812048

**Published:** 2026-03-10

**Authors:** Dong-Wei Di, Yingpeng Hua, Huwei Sun, Sergey Shabala, Yaosheng Wang

**Affiliations:** 1 State Key Laboratory of Soil and Sustainable Agriculture, Institute of Soil Science, Chinese Academy of Sciences, Nanjing, China; 2 University of Chinese Academy of Sciences, Beijing, China; 3 Zhengzhou Research Base, State Key Laboratory of Cotton Bio-breeding and Integrated Utilization, School of Agriculture and Biomanufacturing, Zhengzhou University, Zhengzhou, China; 4 Guangdong Provincial Key Laboratory of Plant Molecular Breeding, Key Laboratory for Enhancing Resource Use Efficiency of Crops in South China, Ministry of Agriculture and Rural Affairs, Guangdong Laboratory for Lingnan Modern Agriculture, College of Agriculture, South China Agricultural University, Guangzhou, China; 5 School of Biological Sciences and ARC Training Centre for Smart and Sustainable Horticulture, University of Western Australia, Crawley, Australia; 6 International Research Centre for Environmental Membrane Biology, Foshan University, Foshan, China; 7 State Key Laboratory of Efficient Utilization of Agricultural Water Resources, Key Laboratory of Dryland Agriculture, Ministry of Agriculture and Rural Affairs of China, Institute of Environment and Sustainable Development in Agriculture, Chinese Academy of Agricultural Sciences, Beijing, China

**Keywords:** efficient utilization, ion transport, membrane transporters, nutrient ion acquisition, stress response

Nutrient ion acquisition, transport, and efficient utilization are fundamental to plant growth, development, and adaptation to changing environments (Schachtman and Shin, 2007; Tan et al., 2022; Di et al., 2024; Mishra et al., 2024; Sharma et al., 2025). Although essential macro− and micronutrients must be absorbed from the soil and distributed within plants, their movement is not a passive process. Instead, it is tightly regulated by networks of membrane transporters, signaling molecules, and post−translational modifications (Shabala et al., 2014; Sharma et al., 2025). Understanding these regulatory networks is crucial for improving crop nutrient use efficiency and stress resilience, key objectives for achieving sustainable agriculture under increasingly challenging environmental constraints (Antony Ceasar et al., 2023). Abiotic stress tolerance was lost during crop domestication (Palmgren et al., 2015; Wang et al., 2020); as a result, crop production across the globe is severely affected by various climate-driven constraints such as drought, heat, salinity, and flooding, costing industry over $180Bln p.a. in lost productivity (Razzaq et al., 2021). Regaining this tolerance is a challenging process that may require a paradigm shift in identifying appropriate breeding targets. Membrane transporters are ideally suited for this purpose.

In this Research Topic, we have compiled a series of reviews and research articles focusing on recent advances in nutrient transport and signaling, with emphasis on nitrogen (N), silicon (Si), carbon (C) allocation, and ion homeostasis under stress conditions. These studies span model plants such as Arabidopsis and rice, as well as economically important species including cucumber and the intertidal macroalga *Pyropia haitanensis*.

## Nitrate signaling integration: from multi−pathway interactions to central signaling hubs

Nitrate (NO_3_^-^) serves not only as a primary N source but also as a key signaling molecule. In this volume, Mao et al. elucidate how NO_3_^-^ signaling integrates with hormones, calcium−dependent phosphorylation cascades (e.g., CBL−CIPK, CPK), reactive oxygen species (ROS), and root−derived peptides (e.g., CEP) to fine−tune root architecture and systemically integrate C−N status. Deeper understanding of this multi−layered network provides actionable targets for improving nitrogen use efficiency (NUE) through genome editing, synthetic chemicals, and engineered nitrogen redistribution (SINAR). Earlier, the rice NO_3_^-^ transporter NRT1.1B has been identified as a dual receptor for nitrate and ABA (Ma et al., 2025). Competitive binding at adjacent sites triggers distinct pathways, promoting growth under high NO_3_^-^ and activating stress responses under low NO_3_^-^ or ABA accumulation. The OsNRT1.1B-OsSPX4-OsNLP4 module therefore functions as a dynamic molecular switch, balancing nutrient acquisition and stress defense and offering precise targets for simultaneously enhancing NUE and stress tolerance.

## Integrated regulatory network of NUE in rice

The molecular regulatory network of NUE in rice was systematically reviewed by Guo et al. As an ammonium (NH_4_^+^)−preferring crop, rice utilizes both NH_4_^+^ and NO_3_^-^, with uptake mediated by the OsAMT and OsNRT/NPF families, respectively. Key regulators, including transcription factors such as OsWRKY23, OsDREB1C, OsGRF4, and OsNGR5, as well as auxin−related OsDNR1, form a signaling network that coordinates N uptake, assimilation, and allocation. NUE differences between *indica* and *japonica* subspecies partly stem from natural variations in genes such as *OsNRT1.1B* and *OsDNR1*. Additionally, pH homeostasis (via *OsNRT2.3b* and *OSA1*) and multi−gene synergies (e.g., *OsAMT1.2*/*OsGS1.2*/*OsAS1*) are crucial for achieving high yield and enhanced NUE. Recent studies have further revealed that high− NH_4_^+^ stress induces NH_4_^+^ toxicity in plants, with futile NH_4_^+^ efflux serving as a key mechanism underlying this toxicity. The magnitude of NH_4_^+^ efflux has been shown to be significantly negatively correlated with both high-NH_4_^+^ tolerance and NUE in rice (Di et al., 2025). Pharmacological or genetic suppression of this efflux enhances NH_4_^+^ uptake and alleviates toxicity, pointing to a tripartite strategy: enhance uptake, optimize assimilation, and suppress efflux (Wu et al., 2025).

## A central hub for carbon allocation and energy balance

Vacuolar sequestration of toxic nutrients requires efficient operation of various tonoplast-based transporters that occur in a cotransport with H^+^. Such H^+^-couples transporters are also critical for high-affinity nutrient uptake by roots and phloem loading and translocation of sugars. Here, Gaxiola and Hirschi systematically review the role of Arabidopsis vacuolar H^+^−pyrophosphatase (AVP1) in these processes. AtAVP1 localizes to both the tonoplast and plasma membrane, where it promotes biosynthesis and enhances sucrose loading and long−distance transport. Its activity is dynamically regulated via ubiquitination (e.g., UBC34−mediated degradation), allowing plants to adapt to metabolic and environmental changes. Overexpression of *AtAVP1* improves biomass, root architecture, and stress tolerance, and natural variants correlate with yield traits. Future strategies should focus on precisely modulating AVP1’s stability, localization, and expression to optimize source-sink carbon allocation and enhance crop resilience.

## Animal−type Na^+^/K^+^−ATPase PhNKA2: a molecular hub for salt tolerance in intertidal red algae

Feng et al. demonstrate that the animal−type Na^+^/K^+^−ATPase PhNKA2 functions as a molecular hub for salt tolerance in the red alga *Pyropia haitanensis*. Expression of *PhNKA2* is strongly induced under high salinity in gametophytes, contrasting with its sporophyte−preferred homolog *PhNKA1*, indicating life−cycle−specific roles. Heterologous expression in *Chlamydomonas reinhardtii* enhances salt tolerance by maintaining K^+^/Na^+^ homeostasis through Na^+^ efflux and K^+^ influx. Yeast two−hybrid analysis revealed interactions with proteins such as PhUsp5, PhMSRB2, PhGDCST, PhDhps, and AC, which likely support salt adaptation through protein stabilization, oxidative protection, and cytoskeletal regulation. This work provides novel targets for engineering salt−resistant crops.

## Active silicon deposition: the role of proline−rich proteins

The role of PRPs in Si biomineralization was investigated by Sun et al. using cucumber as a target species. Among seven *CsPRP* genes, tandem duplicates *CsPRP1* and *CsPRP3* share high evolutionary and expression similarity. Both exhibit strong Si−binding activity under distinct pH conditions and localize polarly in the cell wall. Their expression correlates with tissue Si accumulation, mainly in mature leaves and roots at the seedling stage, expanding to leaves, roots, petals, and stamens at maturity. These findings clarify the mechanistic role of PRPs in Si deposition and support strategies for enhancing crop stress tolerance through Si application.

## Outlook

Collectively, the articles highlight a recurring theme: nutrient movement and stress adaptation are governed by complex, interconnected systems. Transporters are not merely conduits for nutrient movement, but dynamic regulatory components integrated into larger networks involving signal transduction, protein−protein interactions, and metabolic feedback loops. Future research and breeding efforts should move beyond manipulating single genes toward system−level approaches. This may include:

- Elucidating signal crosstalk to deepen our understanding of how nutrient signals (e.g., NO_3_^-^, Si) interact with hormones, ROS, and energy (carbon) status to coordinate whole−plant responses.
- Leverage insights from protein interactomes (e.g., PhNKA2 interactors) and regulatory factors (e.g., UBC34 for AVP1) to enhance the stability, activity, and specificity of key transporters and enzymes.
- Utilize tissue−specific or inducible promoters, gene−editing tools (e.g., CRISPR-Cas9), and synthetic biology to control gene expression and protein function spatiotemporally, minimizing pleiotropic effects.
- Integrating multiple traits by stack genes that improve nutrient uptake (N, Si), efficient C allocation, and robust ion homeostasis (Na^+^/K^+^) to develop crops that simultaneously achieve high yield, enhanced nutrient use efficiency, and resilience to key abiotic stresses.

Abiotic stress tolerance has been present in wild crop relatives but lost during domestication (Palmgren and Shabala, 2024). By integrating molecular insights gained from evolutionary diverse plant systems algae, we can accelerate the design of next−generation crops capable of addressing the dual challenges of global food security and environmental sustainability.
